# Evaluation of hepatitis E antigen kinetics and its diagnostic utility for prediction of the outcomes of hepatitis E virus genotype 1 infection

**DOI:** 10.1080/21505594.2021.1922027

**Published:** 2021-05-18

**Authors:** Mohamed A. El-Mokhtar, Haidi Karam-Allah Ramadan, Muhamad R. Abdel Hameed, Ayat M. Kamel, Sahar A. Mandour, Maha Ali, Mohamed A. Y. Abdel-Malek, Doaa M. Abd El-Kareem, Sara Adel, Eman H. Salama, Khaled Abo Bakr Khalaf, Ibrahim M. Sayed

**Affiliations:** a Department of Medical Microbiology and Immunology, Faculty of Medicine, Assiut University, Assiut, Egypt; b Microbiology and Immunology Department, Faculty of Pharmacy, Sphinx University, Assiut, Egypt; c Department of Tropical Medicine and Gastroenterology, Faculty of Medicine, Assiut University, Assiut, Egypt; d Department of Internal Medicine and Hematology Unit, Assiut University Hospitals, Assiut University, Assiut, Egypt; e Microbiology and Immunology Department, Faculty of Pharmacy, Assiut University, Assiut Egypt; f Department of Microbiology and Immunology, Faculty of Pharmacy, Deraya University, Minia, Egypt; g Department of Medical Biochemistry, Faculty of Medicine, Assiut University, Egypt; h Department of Clinical Pathology, Faculty of Medicine, Assiut University, Egypt; i Department of Clinical Pathology, Faculty of Medicine, Al-Azhar University, Assiut, Egypt; j Department of Clinical Pathology, Faculty of Medicine, Sohag University, Sohag, Egypt; k Department of Pathology, School of Medicine, University of California, San Diego, La Jolla, California, USA

**Keywords:** HEV antigen, HEV genotype 1, acute self-limiting infection, fulminant hepatic failure, HEV kinetic, predictive biomarker

## Abstract

HEV-Ag ELISA assay is a reliable diagnostic test in resource-limited areas. HEV genotype 1 (HEV-1) infections are either self-limited or progress to fulminant hepatic failure (FHF) and death if anti-HEV therapy is delayed. Limited data is available about the diagnostic utility of HEV Ag on HEV-1 infections. Herein wWe aimed to study the kinetics of HEV Ag during HEV-1 infections at different stages, i.e., acute HEV infection, recovery, and progression to FHF. Also, we evaluated the diagnostic utility of this marker to predict the outcomes of HEV-1 infections. Plasma of acute hepatitis E (AHE) patients were assessed for HEV RNA by RT-qPCR, HEV Ag, and anti-HEV IgM by ELISA. The kinetics of HEV Ag was monitored at different time points; acute phase of infection, recovery, FHF stage, and post-recovery. Our results showed that the level of HEV Ag was elevated in AHE patients with a significantly higher level in FHF patients than recovered patients. We identified a plasma HEV Ag threshold that can differentiate between self-limiting infection and FHF progression with 100% sensitivity and 88.89% specificity. HEV Ag and HEV RNA have similar kinetics during the acute phase and self-limiting infection. In the FHF stage, HEV Ag and anti-HEV IgM have similar patterns of kinetics which could be the cause of liver damage. In conclusion, the HEV Ag assay can be used as a biomarker for predicting the consequences of HEV-1 infections which could be diagnostically useful for taking the appropriate measures to reduce the complications, especially for high-risk groups.

## Introduction

Hepatitis E virus (HEV) is a leading cause of acute viral hepatitis globally causing about 14 million infections with 300,000 deaths and 5200 stillbirths worldwide annually [1,2]. HEV is a small icosahedral, positive-sense single-strand RNA virus that includes three to four open reading frames (ORF1-4). ORF1 is located at the 5ʹ end and encodes a nonstructural polyprotein with a methyl transferase, papain-like cysteine protease, RNA helicase, and RNA-dependent RNA polymerase activity [1,3]. ORF2 encodes the structural capsid protein that is involved in HEV entry and modulation of host immune response [4,5]. ORF3 encodes a small cytoskeleton-associated phosphoprotein that is required for the release of infectious HEV particles [6,7]. ORF4 is induced under endoplasmic reticulum stress mainly in HEV genotype 1, and this protein interacts with viral and host proteins to activate the viral replication [8].

HEV isolates that infect humans belong to the *Orthohepevirus* genus of the *Hepeviridae* family. *Orthohepevirus* A includes 8eightgenotypes (HEV 1–8), 5fivegenotypes cause infections in humans [9]. HEV-1 and HEV-2 infect humans in developing countries through the fecal-oral route [10]. HEV-3, HEV-4, and HEV-7 include zoonotic strains and infection is caused by consumption of contaminated raw or undercooked animal products [11,12,13,14,15]. In addition, the transmission of HEV by blood transfusion is documented [16]. Besides, vertical or perinatal transmission of HEV is recorded and usually associated with significant perinatal mortality especially with HEV-1 isolate [17].

HEV infection is an acute self-limiting disease, especially in immune-competent patients. However, progression to fulminant hepatic failure (FHF) was recorded [18,19]. HEV-3 and HEV-4 infections may develop chronicity in immunocompromised patients such as HIV infected, leukemic, and organ transplant patients [20,21]. The diagnosis of HEV infection is based mainly on the detection of HEV RNA (gold standard), detection of anti-HEV antibodies (IgM and/or IgG), and/or the detection of HEV ORF2 antigen (Ag) [22,23,24]. Previous studies showed that the HEV Ag ELISA could be used as a reliable diagnostic tool in clinical laboratories where molecular assays are lacking [22,25,26,27,]. In addition, HEV Ag can differentiate between acute and chronic HEV-3 infections [28], and the serum level of HEV Ag could predict the possibility of HEV chronicity in the immunocompromised patient [29]. Limited data is available about the diagnostic utility of HEV Ag on HEV-1 infections. The fate of acute HEV infection (AHE) caused by HEV-1 infection is either self-limiting disease or progression to FHF which causes morbidity if the anti-HEV therapy (i.e., ribavirin) starts late [30].

Herein we aimed to study the kinetics of HEV Ag during HEV-1 infections at different stages, i.e., AHE, recovery, and progression to FHF. Also, we evaluated the diagnostic utility of this marker to predict the outcomes of HEV-1 infections.

## Material and methods

### Study population

Patients with clinical symptoms of acute hepatitis admitted to outpatient Cclinics, Internal Medicine Department, and Tropical Medicine and Gastroenterology dDepartments of Assiut University Hospital, Assiut Fever Hospital, AL-Rajhi Liver University Hospital, Al-Azhar University Hospital, and Sohag University Hospital, Egypt during the period from January 2019 till December 2020 were recruited (Table 1). Acute hepatitis E patients were presented with one or more of the clinical manifestations of acute hepatitis symptoms such as fever, jaundice, dark urine, pale stool, and abdominal pain. Fulminant Hepatic Failure (FHF) patients in this study were developed severe liver injury such as ascites, coagulopathy (INR ≥1.5), and hepatic encephalopathy within 1–8 weeks of the illness. More details are reported in supplementary material and methods. All participants provided written informed consent, and the protocol of HEV detection in the blood samples was approved by the Institutional Review Board (IRB no 17200190) at the Faculty of Medicine, Assiut University, Egypt, in accordance with the provisions of the Declaration of Helsinki.

**Table 1.** Patients Criteria enrolled in the study

**Patient ID**	**Sex** **M/F**	**Age, y**	**Acute phase of infection**								**Outcome**	**Mortality**	**Therapy**^d^	**Hospitalization** **(Days)**
			**ALT** **U/l**	**AST** **U/l**	**ALP** **U/l**	**Bilirubin** **µmol/L**	**INR**	**Anti-HEV IgM**^a^	**HEV RNA**	**HEV Ag** **S/CO**^c^				
1	M	66	1100	780	420	450	1.9	+	+	+	FHF	Yes	supportive	28
2	M	60	950	680	510	540	1.8	+	+	+	FHF	Yes	supportive	24
3	F	72	1040	720	310	380	2	+	+	+	FHF	Yes	supportive	44
4	F	60	880	580	280	420	2.4	+	+	+	FHF	Yes	supportive	56
5	F	76	960	640	240	240	1.3	+	+	+	Self-limiting	No	supportive	33
6	M	66	860	580	320	280	2.5	-	-^b^	+	FHF	Yes	supportive	48
7	M	72	120	95	140	230	1.4	-	+	+	Self-limiting	No	supportive	38
8	F	44	124	110	400	180	1.2	+	+	+	Self-limiting	No	supportive	19
9	M	57	240	134	500	220	1.3	+	+	+	Self-limiting	No	supportive	21
10	F	55	310	240	110	190	1.2	+	+	+	Self-limiting	No	supportive	14
11	M	51	209	130	400	150	1.4	-	+	+	Self-limiting	No	supportive	15
12	M	48	953	420	320	190	1.3	+	+	+	Self-limiting	No	supportive	13
13	F	63	956	430	380	440	2.6	-	- ^b^	+	FHF	Yes	supportive	30
14	M	50	944	250	270	220	1.1	+	+	+	Self-limiting	No	supportive	21
15	F	22	134	88	256	245	1.2	+	+	+	Self-limiting	No	supportive	14
16	M	37	340	234	360	256	1.4	-	+	+	Self-limiting	No	supportive	18
17	F	44	240	160	220	200	1.2	+	+	+	Self-limiting	No	supportive	20
18	M	55	340	220	110	310	1.1	+	+	-	Self-limiting	No	supportive	17
19	F	60	220	405	140	200	1.3	+	+	-	Self-limiting	No	supportive	9
20	M	76	210	106	260	340	1.3	+	+	-	Self-limiting	No	supportive	35
21	F	66	240	160	150	330	1.2	+	+	-	Self-limiting	No	supportive	29
22	M	72	200	105	188	345	1.1	-	+	-	Self-limiting	No	supportive	36
23	F	44	950	410	179	290	1.1	+	+	+	Self-limiting	No	supportive	16
24	M	57	845	520	120	190	1.4	+	+	+	Self-limiting	No	supportive	10

ALT, alanine aminotransferase; AST, aspartate aminotransferase; ALP, Alkaline phosphatase; INR, international normalized ratio; +, positive, -, negative, S/CO, signal (OD_450/630_) to cut off, FHF: fulminant hepatic failure.

^a^The value of S/CO for each specimen >1.1 indicates positive for anti-HEV IgM, and a value of <0.9 indicating negative.

^b^below the limit of quantification (LOQ), < 300 IU/ml.

^c^The value of S/CO for each specimen >1.1 indicates positive for HEV Ag, and a value of <0.9 indicating negative.

^d^supportive therapies include hepatoprotective agents such as Silymarin, ursodeoxycholic acid, and cholestyramine. Vitamin K, fresh frozen plasma, Lactulose and + Rifaximine+ (L-ornithine+L aspartate) were given to FHF patients. No antiviral therapies such as RBV and/or IFN were given to the patients enrolled in this study.

### Assessment of HEV Ag and anti-HEV IgM

The level of HEV Ag and anti-HEV IgM were tested in the patients’ plasma samples using Wantai ELISA kits (Wantai Biologic Pharmacy Enterprise, Beijing, China) according to the manufacturer’s instructions with slight modification (Details in supplementary).

### Molecular detection of HEV RNA and sequencing

HEV RNA was extracted from the plasma of AHE patients using QIAamp Viral RNA Mini Kit (Qiagen, Germany). HEV RNA was quantified by qRT-PCR using primers targeting HEV ORF2/3 region as described previously [31,32,33]. Nested PCR and sequencing were done on the isolated viruses using primers targeting HEV ORF2 as previously described [31,33,34].

### Statistics

Statistical analyses were performed using the GraphPad Prism software 8 (GraphPad Software, La Jolla, USA). Results are expressed as mean ± SEM, median with interquartile range unless otherwise specified. Correlation analyses were calculated with the Spearman’s rank correlation coefficients. The correlation coefficients of r > 0.4 with significance statistic (p < 0.05) were considered strong positive. Comparisons of different groups was performed using a Bonferroni-corrected multiple comparisons t test. *P* < 0.05 was considered significant. Receiver operating characteristic (ROC) curve was plotted, a putative threshold was calculated.

## Results

### Assessment of HEV markers in acute hepatitis E (AHE) patients

**Figure 1.**
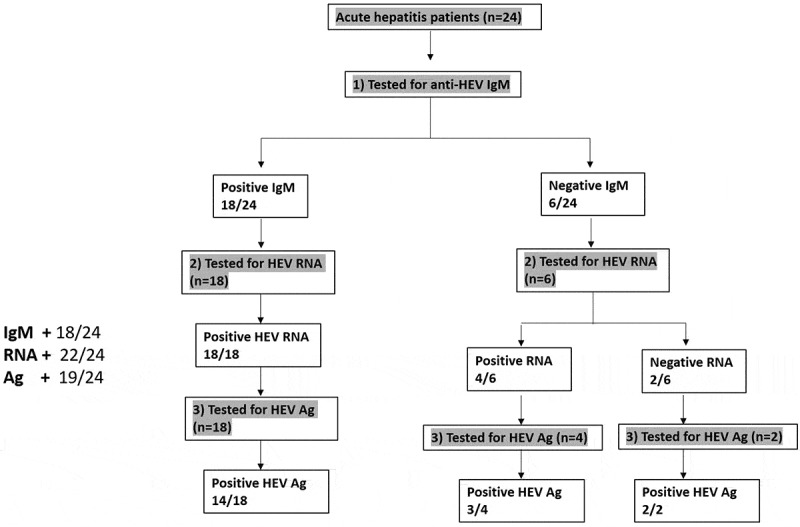
**Assessment of HEV markers in AHE patients**. Acute hepatitis patients (n = 24) were screened for HEV markers (anti-HEV IgM, HEV RNA, and HEV Ag). Eighteen samples were reactive to anti-HEV IgM and HEV RNA, and 14 out of 18 samples were also positive to HEV Ag. While 6 samples were negative for anti-HEV IgM, four out of the 6 samples were positive for HEV RNA, and three out of these four samples were also positive for HEV Ag. Two out of the 6 samples were negative for anti-HEV IgM and HEV RNA, while HEV Ag was positive in these samples

Twenty-four patients were diagnosed with acute HEV infection (AHE) based on the detection of anti-HEV IgM, HEV RNA, and/or HEV Ag (Figure 1) according to the guideline of EASL [23]. Overall, the detection rate was 91.6%, 75%, and 79.1% for HEV RNA, anti-HEV IgM, and HEV Ag, respectively. Eighteen samples (n = 18) were positive for anti-HEV IgM and HEV RNA, from which 14 samples (14/18, 78%) tested positive for HEV Ag. Out of 24 samples, 6 samples tested negative for anti-HEV IgM, while four out of 6 samples tested positive for HEV RNA and HEV Ag was detectable in 3 out of 4 of HEV RNA positive samples (Figure 1). Two samples (2/24, 8.33%) were negative for anti-HEV IgM and HEV RNA, while HEV Ag was detectable (Figure 1).

### HEV Ag correlation to the plasma viral load and liver enzymes during AHE infection

**Figure 2.**
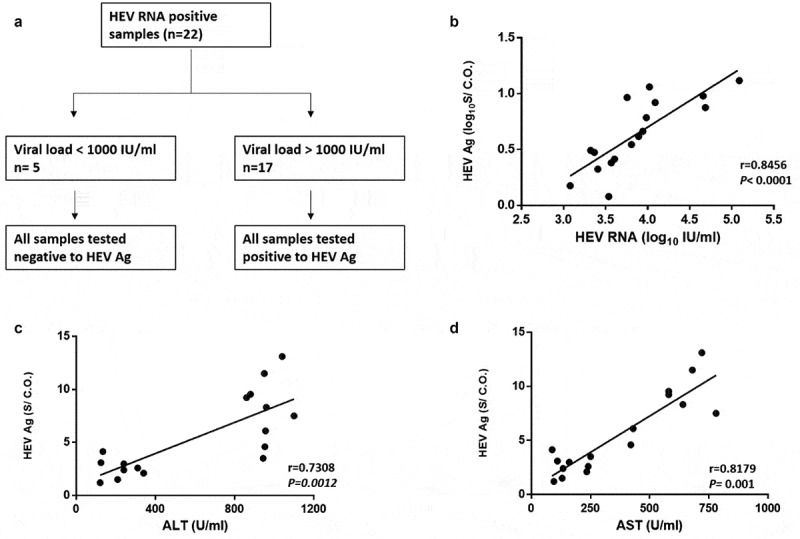
**HEV Ag is correlated to the plasma viral load and liver enzymes**. (a) HEV RNA positive plasma samples (n = 22), 17 out of 22 were positive to HEV Ag, all these samples have a viral load > 10^3^ IU/ml. While 5 out of 22 samples were negative to HEV Ag, all these samples have a viral load < 10^3^ IU/ml. (b) Correlation between HEV Ag (log_10_S/CO) and HEV RNA (log_10_IU/mL) in the plasma (r = 0.8456; *P* < 0.0001; n = 17). (c) Correlation between HEV Ag (S/CO) and ALT (U/ml) in the plasma (r = 0.7308; *P* = 0.0012; n = 17). (d) Correlation between HEV Ag (S/CO) and AST (U/ml) in the plasma (r = 0.8179; *P* = 0.001; n = 17)

Twenty-two samples out of 24 samples were positive for HEV RNA during the acute phase of infection (Figure 1). The viral load was assessed in HEV RNA positive samples, and the median (interquartile range, IQR) of plasma HEV RNA load was 3.586 (3.053–3.994 log_10_ IU/ml). Sequencing was successful in 15 samples, and all of them belonged to HEV genotype 1 subtypes 1b (n = 8) and 1e (n = 7). Out of 22 HEV RNA positive plasma samples, 17 samples (77.27%) tested positive for HEV Ag, while 5 samples (22.73%) were negative for HEV Ag. All the samples tested negative for HEV Ag have a viral load less than 3 log_10_ IU/ml, while HEV Ag positive samples have a viral load higher than 3 log_10_ IU/ml (Figure 2a). The median value with IQR of plasma HEV Ag was 4.14 (2.5–8.78) S/CO and there was a correlation between plasma HEV Ag and plasma HEV RNA (r = 0.8456, *P* < 0.0001, n = 17) (Figure 2b). Also, the plasma HEV Ag was correlated to the level of plasma liver enzymes such as ALT (r = 0.7308, *p = 0.0012*, n = 17) (Figure 2c) and AST (r = 0.8179, *p = 0.0001*, n = 17) (Figure 2d).

### The plasma HEV Ag level at the acute phase of infection can predict the outcomes of HEV-1 infection

**Figure 3.**
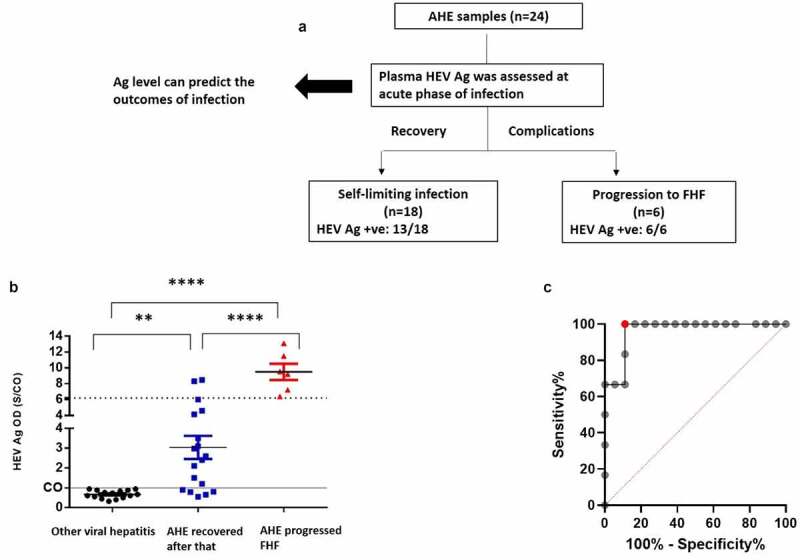
**The plasma HEV Ag can predict the outcome of acute HEV-1 infection**. (a) Plasma HEV Ag was assessed at the acute phase of infection in AHE patients (n = 24) and linked with the outcomes of infections. 18 out of 24 patients were recovered, from which 13/18 were HEV Ag were positive at the acute phase of infection. 6 out of 24 patients were progressed to FHF, all of them were positive to HEV Ag at the acute phase of infection. (b) The plasma level of HEV Ag (S/CO) was assessed in non-HEV acute viral hepatitis patients (black), AHE patients who recovered after that (blue), and AHE patients who progressed to FHF patients (Red). This assessment was done during acute phase of infections (time of hospital admission). The dashed line indicates a putative S/CO threshold (6.05) for distinguishing patients with cleared the infection from whom progressed to FHF as calculated in panel C. Differences in means were tested with a Bonferroni corrected t test. **, **** mean *P* < 0.01 and 0.001, respectively. (c) Receiver operating characteristic curve showing the true positive rate plotted against the false positive rate at different plasma HEV Ag threshold values to differentiate patients according to the outcomes of infection. The red circle represents 100% sensitivity 95% CI, [54.07%-100%]) and 88.89% specificity (95%CI [65.29%-98.62%]) at an HEV Ag value of 6.05 (AUC: 0.9630, 95%CI [0.8931–1.033]; likelihood ratio, 9, *P* = 0.0008644)

In this cohort, 18 out of 24 patients (75%) were recovered spontaneously (self-limiting infection) and 6 patients (25%) were progressed to FHF. HEV Ag was assessed in the plasma of these patients at acute phase of infection, and we correlated the Ag level with the outcomes that developed after that. We detected HEV Ag in the plasma of 13/18 (72.22%) of the recovered patient, while HEV Ag was detected in 6/6 (100%) of the patients who developed FHF at the acute phase of infection. We assessed if the plasma level of HEV Ag at acute phase of infection can predict the outcomes of HEV-1 infection (Figure 3a). First, we assessed the specificity of this assay against other viral hepatitis samples such as HCV (n = 4), HBV (n = 4), HAV (n = 3), CMV (n = 2), and EBV (n = 2), and we found that the specificity of this assay is 100% with no ccross-reactivitywith other viral hepatitis (Figure 3b).

The level of HEV Ag (expressed S/CO values) was significantly elevated in acutely self-limiting and FHF patients, compared with non HHEV-infectedcontrols. Patients who progressed to FHF had a significantly higher plasma HEV Ag level (median with IQR of S/CO: 9.385 [7.023–11.9]) than those who spontaneously cleared the virus (2.5 [0.87–4.255]; p < 0.0001) at the acute phase of infection (Figure 3b). ROC curve was plotted to identify the plasma HEV Ag threshold that differentiates between acute self-limiting infection and FHF progression (Figure 3c). An S/CO OD threshold of 6.05 discriminated between self-limiting patients and FHF patients with a sensitivity of 100% (95% confidence interval [CI], [54.07%-100%]) and specificity was 88.89% (95%CI [65.29%-98.62%]) (area under the curve (AUC): 0.9630, 95%CI [0.8931–1.000]; likelihood ratio, 9, *P* = 0.0009) (Figure 3c).

### Kinetic of HEV Ag during self-limiting infection of HEV −1 and follow up study

**Figure 4.**
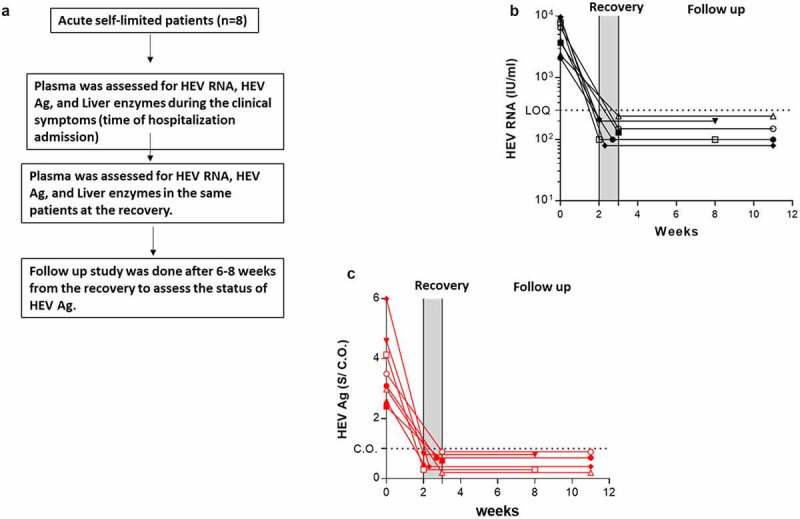
**Kinetic of HEV Ag during self-limiting infection of HEV −1 and follow up study**. (a) Flow chart of the study design. Plasma samples from AHE patients (n = 8) were assessed for HEV RNA (black) (b) and HEV Ag (red) (c) at the time of acute infection and recovery. Plasma samples from recovered patients (n = 6) were assessed for the same markers 6–8 weeks post recovery (b, c). The same symbol in (B, C) indicates the same patient, and different color means different marker as described. LOQ: limit of quantification, CO: cut off, and S/CO: signal to cut off

We assessed the level of HEV Ag and its correlation to HEV RNA and liver enzymes during HEV clearance (self-limiting infection). Samples from eight patients were available at different time points (i.e., acute phase and recovery). At the recovery, liver enzymes were returned to normal levels and HEV RNA was cleared from the plasma (Figure 4a, 4b). The loss of HEV RNA in the recovery period was accompanied by a parallel loss of HEV Ag in those patients and HEV Ag was under the CO (Figure 4c). A follow-up study was done, and the status of HEV Ag and HEV RNA was monitored in 6 patients after 6–8 weeks of recovery. HEV Ag and HEV RNA were not recorded in the plasma of these patients (Figure 4b, 4c).

### Kinetic of HEV Ag in the patients developed FHF and its correlation to HEV markers

**Figure 5.**
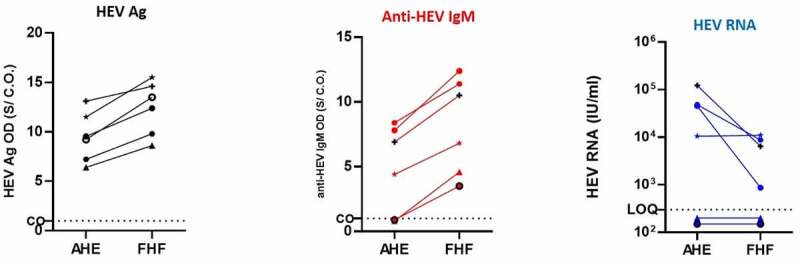
**Kinetic of HEV Ag in the patients developed FHF and its correlation to HEV markers**. Plasma samples from AHE patients who progressed to FHF (n = 6) were assessed for HEV Ag (black) (a), ant-HEV IgM (red) (b), and HEV RNA (blue) at the time of acute infection and FHF development. The same symbol in (A, B, and C) indicates the same patient, and different color means different marker as described. LOQ: limit of quantification, CO: cut off, and S/CO: signal to cut off

In this cohort, 6 out of 24 patients (25%) were progressed to FHF within 1–8 weeks of hospital admission (acute phase of infection). We assessed the kinetics of plasma HEV Ag at two different time points: acute phase of infection (hospital admission) and at the stage of FHF and its correlation to other HEV markers. During FHF progression, the level of HEV Ag was increased in the patients alongside the elevation of liver enzymes and anti-HEV IgM (Figure 5a, 5b). Anti-HEV IgM was not detectable (below CO) in two patients during the AHE infection, and the level of anti-HEV IgM was elevated in those patients in the FHF stage (Figure 5b).

On the other hand, the viral load was either unchanged or decreased during FHF progression (Figure 5c). HEV RNA was under LOQ in two patients during the AHE stage, and the viral load was also no detectable in the FHF stage. In the other four patients, the viral load was decreased in the FHF stage in three patients and the viral load remained at the same level in the fourth patient (Figure 5c).

## Discussion

HEV Ag assay is a reliable diagnostic tool, and it is recommended in clinical settings where the molecular diagnostic approach is not available [22,23,35]. Besides, HEV Ag can be used as a diagnostic tool in the window period before seroconversion and in settings where seroconversion may be delayed or absent such as immunosuppression [22,26,28,36,37]. HEV-Ag assay showed superior performance than anti-HEV IgM for diagnosis of acute HEV-3 infection in immunocompromised patients [22]. In addition, HEV Ag can differentiate between acute and chronic HEV-3 infections and can be a useful predictive marker for the evolution of chronicity [28,29]. Furthermore, the kinetic of HEV Ag during chronic HEV infections and its impact on HEV diagnosis was studied in human liver chimeric mice [35]. Long-term HEV viremia and/or antigenemia and without a serologic response or symptoms of AHE were recorded in immunocompetent blood donors [38,39]. However, limited data is available on the diagnostic utility of the HEV Ag assay on HEV-1 infections. In this study, we assessed the performance of the HEV Ag assay in acute HEV-1 infections, and we monitored the kinetics of HEV Ag during the acute phase of infection and the outcomes. We assessed the possibility of using the HEV Ag as a predictive marker for HEV-1 consequences. Up to our knowledge, this is the first study that reports the diagnostic utility of HEV Ag to predict the outcomes of HEV-1 infections.

In this study, HEV RNA was detectable in 22/24 of AHE patients (91.6%), sequencing results showed that the isolated viruses belonged to HEV-1. While HEV Ag was detectable in 19/24 of AHE patients (79.15%), and 17 out of 22 HEV RNA positive sera (77.27%), HEV Ag was not detectable in samples with low viral loads (HEV RNA <1000 IU/ml) suggesting that the HEV Ag assay is less sensitive than qPCR in the detection of HEV-1 infections. Similarly, Behrendt et al. showed HEV RNA levels of <10 000 copies/mL led to negative test results of HEV Ag ELISA assay [28]. Also, Trémeaux et al. reported that HEV Ag assay can detect AHE plasma caused by HEV-3 with HEV RNA concentrations ranged from 800 to 80,000 IU/ml [22]. While two samples in this study were positive for HEV Ag but negative for HEV RNA and anti-HEV IgM, we hypothesized that the time of sample collection (hospital admission) or the risk of FHF progression (as will be described below) could explain this result. Majumdar et al. assessed the kinetics of HEV markers in the sera of AHE patients during an outbreak caused by HEV-1, and they reported that HEV Ag and HEV-RNA assays showed 100% positive results in the first 3 days of illness, but the positive HEV RNA declined to 54% by days 4–7, whereas HEV antigen and anti-HEV IgM was 88% and 100% positive during this period [37]. HEV Ag was detected in the early phase of infection where anti-HEV IgM was negative [36,37]. Likewise, Behrendt et al. reported that HEV capsid structures without HEV RNA content can be found at different densities of sera from HEV-3 infected patients [28]. In a parallel line, using *in vivo* humanized mouse model, Sayed et al. reported that HEV Ag could be detected earlier than HEV RNA in the plasma of some, not all, HEV-1 and HEV-3 infected mice [35]. In addition, Montpellier et al. and Sayed et al. reported the presence of high levels of noninfectious ORF2 Ags in human and mouse plasma, respectively, and these Ags were the target of the HEV-Ag ELISA assay, this could also explain the presence of HEV Ag in RNA negative samples [35,40]. Likewise, Yin and colleagues showed the release of large amounts of nonvirion-associated ORF2 Ag in the patient sera and cell culture supernatants during HEV infection, which is different from the actual viral capsid protein [41]. Collectively, our results suggested that though HEV RNA is the gold standard of HEV diagnosis and more sensitive than HEV Ag ELISA assay in most cases. However, HEV Ag assay could be the only diagnostic markers in certain circumstances, especially if the clinical symptoms and laboratory results (exclusion of other causes of viral hepatitis) confirm HEV diagnosis.

In this study, we showed that the specificity of the HEV Ag ELISA assay is 100%, and there was no cross-reactivity with other viral hepatitis viruses such as HAV, HBV, HCV, CMV, and EBV. Similarly, Trémeaux et al. and Behrendt et al. showed that the specificity of this assay is 100% and 92%, respectively [22,28]. Our result showed that the level of HEV Ag was correlated to the plasma viral load (r = 0.8456, *P* < 0.0001) and the level of liver enzymes (ALT and AST, r = 0.7308 and r = 0.8179, respectively) during the acute phase of infection. Likewise, Gupta et al. showed that HEV Ag, but not anti-HEV IgM, had a good concordance with HEV RNA [25]. Besides, Marion and colleagues showed that serum HEV Ag, but not urinary HEV Ag, was correlated to serum HEV RNA during the acute phase of HEV-3 infection in solid organ transplants [29]. In this study, we assessed if the acute phase serum HEV Ag level can predict the outcome of HEV-1 infections. HEV-1 infected patients either cleared the virus spontaneously (self-limiting) or progressed to severe complications such as FHF. Up to our knowledge, HEV-1 infections have not been developed to chronicity. In the acute phase of infection, HEV Ag was detected in 13 out of 18 patients (72.22%) who recovered spontaneously after that, while HEV Ag was detected in 100% of patients who progressed to FHF. The level of HEV Ag was significantly higher in FHF patients than self-limited patients during acute infection. HEV Ag is a relevant marker of active HEV replication and the production of HEV Ag is cumulative [25,35]. Therefore, the level of HEV Ag is increased with ongoing infection in a time-dependent manner, this could explain the higher level of HEV Ag in FHF patients than recovered patients. ROC analysis indicated that the acute phase plasma HEV Ag discriminated between the two groups and an HEV Ag threshold of >6.05 was associated with 100% sensitivity, 88.89% specificity, and a 9-fold increase in the likelihood ratio of an FHF progression. The Ag threshold value in our study reflects the test results of this small cohort and might differ at other sites depending on the patient criteria, methodology procedure, and instruments. Behrendt et al. previously suggested that HEV ORF2 Ag levels can differentiate between acute and chronic HEV Infection of genotype 3; and the likelihood of chronic infection was increased 8-fold when the serum level HEV Ag was >15.76 (Behrendt et al., 2016). Another study reported that serum HEV Ag discriminated between the acute and chronic HEV-3 infection and a log_10_S/CO HEV Ag threshold >3.56 was associated with 80% sensitivity and 100% specificity [29]. Up to our knowledge, this is the first study that reports the utility of HEV Ag assay in predication the outcomes of HEV-1 infections and progression to FHF.

In this study, we assessed the kinetics of HEV Ag during the viral clearance and progression to FHF in the setting of HEV-1 infections. In acute self-limiting infection, the decrease of HEV Ag and HEV RNA levels was concomitant, and they were accompanied with the restoration of the liver function tests to normal levels. A follow-up study confirmed the finding and HEV RNA and HEV Ag were not detectable in the plasma of recovered patients 4–8 weeks post-recovery. Unfortunately, we could not collect stool samples from the recovered patients to test HEV markers since the protocol of HEV diagnosis is not routinely done in Egypt. Similarly, HEV Ag and HEV RNA were concomitantly reduced during acute self-limiting HEV-3 infection [28] . In acute HEV-4 infection, HEV Ag became undetectable 4 weeks earlier than HEV RNA [36]. On the other hand, HEV Ag remained detectable for 60–200 days in chronic HEV-3 infection after ribavirin therapy and HEV RNA clearance [28]. During AHE infection to FHF progression, the level of HEV Ag was increased in six patients parallel to the increase of anti-HEV IgM, while the overall viral load was decreased. Likewise, Saravanabalaji et al. reported that FHF patients had significantly higher anti-HEV IgM titer than recovered patients [42]. Also, our previous study on another Egyptian cohort showed that the level of anti-HEV IgM was significantly higher in FHF patients than recovered patients in the acute phase of infection [43]. The high levels of plasma HEV Ag and anti-HEV IgM in FHF patients could explain the mechanisms of HEV induced liver injury. Since HEV is not a hepatotoxic virus, HEV-associated liver injury is mediated by host immune mechanisms which involve the excessive immune response against the viral antigens, deposition of the immune complexes, and the infiltration of the inflammatory cells around the deposits [44].

The decrease of HEV RNA during FHF progression suggests that the liver damage may not be associated with excessive HEV replication. Similarly, previous reports showed that HEV RNA was not detectable in FHF patients and higher viral load was recorded in recovered patients compared to FHF patients [42,43,45]. It was not possible to obtain liver biopsies from FHF patients, and therefore the possible replication of HEV in the liver during the FHF stage could not be ascertained. Contrary to our finding, Kar et al. reported that the viral load was significantly higher in FHF pregnant patients than AVH pregnant women [46]. Collectively, our results showed that the kinetics of HEV Ag and HEV RNA are in the same direction in the acute phase of infection, but this is not the case in the stage of FHF. In the FHF stage, HEV Ag and anti-HEV IgM have similar patterns of kinetics which cause liver damage. Further studies need to ascertain our findings.

In this study, FHF patients were older and required a longer period of hospitalization than the recovered patients. Age and hematological malignancies were the risk factors for FHF development in this cohort. Similarly, previous reports showed that Age and hematological malignancies were prognostic markers for the complications of HEV infection [18,43,47]. Also, longer hospitalization periods were required for FHF patients [18,43].

Our results showed that the plasma level of HEV Ag assay in the acute phase of HEV-1 infection can predict the risk of FHF progression. Clinicians can benefit from the diagnostic utility of this assay to predict the consequences of the HEV-1 infection and take the appropriate curative and preventive measures to reduce the complications especially for high-risk groups such as old ages, and patients receiving immunosuppressive and chemotherapies.

One limitation of this study is the small number (n = 24) of the subjects in our cohort. However, the percentage of FHF development in this cohort is 25% (6/24) which is higher than the previously reported percentage (5–15%) of HEV-developed acute live failure [43,48,49]. Although we believe that our cohort is unique, further studies, including larger numbers of HEV-1 infected patients need to ascertain our findings. Also, the patients enrolled in this study were infected with HEV only. Coinfection of HBV or HCV with HEV is reported and associated with more severe complications [50,51]. It is worthy to study the kinetics of HEV Ag in coinfected cohort and the predictive approach of the HEV Ag assay in the settings of coinfection. Moreover, none of the enrolled subjects in this study was pregnant. HEV-1 causes severe complications and high mortality (up to 30%) during pregnancy, especially in the third trimester [17,52]. Further studies need to assess the diagnostic level of HEV Ag to predict the outcomes of HEV-1 infection in pregnant women. Likewise, extrahepatic manifestations are associated with HEV infection [44,53,54,55,56,57], this diagnostic assay could be also beneficial to predict the possibility of extrahepatic disorders associated with HEV infection.

In conclusion, we show the performance of the HEV Ag assay during HEV-1 infections. HEV Ag could be the only diagnostic marker especially if the clinical and laboratory results support HEV diagnosis. High HEV Ag levels in the acute phase of infection could reflect higher HEV replication in the liver but do not necessarily associate with liver disease. Also, we report for the first time the kinetics of HEV Ag in AHE, recovery, and FHF progression. We present the diagnostic utility of this assay to predict the outcomes of HEV-1 infections which could be diagnostically useful for taking the appropriate measures to reduce the complications, especially for high-risk groups.

## Supplementary Material

Supplemental Material

## Data Availability

The data that support the findings of this study are available from the corresponding author, upon reasonable request.
