# Supplementary material for: Evaluation of hepatitis E antigen kinetics and its diagnostic utility for prediction of the outcomes of hepatitis E virus genotype 1 infection
Source: Virulence. 2021 May 18;12(1):1334–44. doi: 10.1080/21505594.2021.1922027 (PMC8143225; doi:10.1080/21505594.2021.1922027)
Supplement: Supplemental Material [file KVIR_A_1922027_SM7319.docx]

**Supplementary material and Methods**

**1- Study Population**

The patients presented with one or more of the clinical manifestations of acute hepatitis symptoms such as fever, jaundice, dark urine, pale stool, and abdominal pain. Patients with liver disease of known etiology and evidence of chronic liver diseases were excluded from the study. Blood samples were collected from the patients and screened for the following: liver function tests (LFTs) including liver transaminases (alanine transaminase (ALT) and aspartate transaminase (AST), and routine viral hepatitis markers (HAV, HBV, HCV, CMV, and EBV).

**2- Screening of acute viral hepatitis (AVH)**

Screening for HAV and CMV was performed using a rapid test for anti-HAV IgM (CTK Biotech, San Diego, CA, USA) and anti-CMV-IgM (MyBioSource, Inc. San Diego, USA), respectively. HCV diagnosis was assessed for anti-HCV IgG using the fourth generation HCV Tri-Dot test control (Atlas Link, USA) and HCV RNA by qPCR. Screening of HBV infection was performed on HBV DNA by qPCR, and the detection of HBsAg (ACON laboratories, Inc., USA), and anti-HBV core IgM (IND Diagnostic, Delta, Canada). EBV screening was on done by the monospot test. The diagnosis of HEV infection is not routinely done in Assiut hospitals.

**3- Assessment of HEV Ag and anti-HEV IgM**

The level of HEV Ag and anti-HEV IgM was tested in the patients’ plasma samples using Wantai ELISA kits (Wantai Biologic Pharmacy Enterprise, Beijing, China) according to the manufacturer’s instructions with slight modification (Details in supplementary). Briefly, we included plasma samples collected from healthy Egyptian subjects besides the negative control (NC) provided by the kit, to confirm the accurate cut off (CO). The ratio of the OD450/630 value for each individual sample to the CO (S/CO) was used to indicate HEV Ag and anti-HEV IgM status. S/CO > 1.1 was considered positive and S/CO < 0.9 was considered negative.
